# Smokers’ unprompted comments on cigarette additives during conversations about the genetic basis for nicotine addiction: a focus group study

**DOI:** 10.1186/s12889-018-5395-8

**Published:** 2018-04-13

**Authors:** Sydney E. Philpott, Sarah Gehlert, Erika A. Waters

**Affiliations:** Division of Public Health Sciences, Washington University in St. Louis School of Medicine, St. Louis, Missouri USA

## Abstract

**Background:**

Research designed to elicit smokers’ cognitive and affective reactions to information about chemicals that tobacco companies add to cigarettes (“additives”) found that knowledge is limited. However, little is known about smokers’ unprompted thoughts and feelings about additives. Such information could be used to shape future communication efforts. We explored the content and possible functions of spontaneous statements about cigarette additives made by smokers during a study examining reactions to learning about the genetic link to nicotine addiction.

**Methods:**

Adult smokers (*N* = 84) were recruited from a medium-sized Midwestern city. Focus groups (*N* = 13) were conducted between April-September 2012. Data were analyzed by 2 coders using thematic analysis.

**Results:**

Comments about cigarette additives arose without prompting by the focus group moderator. Three main themes were identified: (1) discussing additives helped participants navigate the conceptual link between smoking and genetics, (2) additives were discussed as an alternative mechanism for addiction to cigarettes, and (3) additives provided an alternative mechanism by which cigarette smoking exacerbates physical harm. Notably, discussion of additives contained a pervasive tone of mistrust illustrated by words like “they” and “them,” by statements of uncertainty such as “you don’t know what they’re putting into cigarettes,” and by negative affective verbalizations such as “nasty” and “disgusting”.

**Conclusions:**

Participants had distinct beliefs about cigarette additives, each of which seemed to serve a purpose. Although mistrust may complicate communication about the health risks of tobacco use, health communication experts could use smokers’ existing beliefs and feelings to better design more effective anti-smoking messages.

## Background

Dozens of toxic chemicals are either added to cigarettes or produced during tobacco combustion [1]. Many of these compounds are carcinogenic [2–6], and some chemicals are added to increase product appeal, ease smoking initiation, discourage cessation, or promote relapse [7].

There are 93 known potentially harmful chemicals in cigarettes [8]. “Constituents” are by-products of the tobacco growing and manufacturing process or result from the combustion processes. “Additives” refer to non-tobacco substances that a manufacturer introduces into the tobacco, paper, or filter [9]. Many studies have examined knowledge, perceptions, and beliefs about cigarette additives and constituents. In general, laypeople are aware that tobacco companies add chemicals to cigarettes or tobacco [10]. While people tend not to know the quantity of chemicals added to cigarettes [11] they are suspicious of additives [12–15]. However, they are less aware of tobacco constituents [14, 16, 17], in some cases erroneously believing that “natural” cigarettes are safe [18–21].

Current literature on perceptions of cigarette additives uses a reactive approach, in which participants are asked to respond to questionnaire items [11, 15] or to indicate their familiarity with specific chemicals [14, 16, 22, 23]. However, it may be useful to assess participants’ spontaneous, unprompted thoughts about additives. Spontaneous thoughts may reflect smokers’ existing beliefs and ways of thinking (or “mental models”) about additives and their role in the harms associated with tobacco use. These mental models, in turn, could shape how smokers interpret tobacco-related information [24–26].

Spontaneous thoughts may also provide unanticipated insights into smokers’ beliefs about the health effects of smoking. For example, spontaneous comments may yield information into how smokers’ thoughts and beliefs about additives relate to their smoking behaviors. Alternatively, they may provide insight into how smokers think about the biological processes, like genetics, that link smoking to specific health consequences. Understanding how smokers think about these biological mechanisms that can shape behavior may lead to more effective anti-smoking messages [27]. Finally, spontaneous utterances may be less vulnerable to social desirability than beliefs assessed via self-reported questionnaire.

Our objective was to explore links among smokers’ spontaneous comments about cigarette additives, their beliefs about the identity and characteristics of such additives, and their potential health consequences. We examine only “additives” because participants never mentioned constituents.

## Methods

All study materials and procedures were approved by the Washington University IRB.

We conducted a secondary analysis of focus group data collected in April-September 2012 to examine smokers’ beliefs about the relationship between genetics and nicotine addiction. Full details are reported elsewhere [25, 28].

### Study sample

Eligible participants were ≥ 18 years old, were current cigarette smokers, self-identified as African American or White, did not consider themselves genetic experts, had attended to the news at least once in the past week, demonstrated rudimentary knowledge of the terms “gene” or “genetic,” and spoke and read English [25, 28]. Participants were recruited from the community through flyers, word of mouth, and a volunteer research participant registry.

### Participant characteristics

Focus groups were stratified by race and education, resulting in the following strata: higher education/African American (HA, *n* = 4), lower education/African American (LA, n = 4), higher education/White (HW, *n* = 2), and lower education/White (LW, *n* = 3) [25, 28]. Focus group size averaged six participants with a range of three to ten. Of 84 participants, 41 (49%) were women, 57 (68%) had less than a Bachelor’s degree, and 52 (62%) identified as African American. Average age was 42.8 (SD = 12.9) and 95.9% smoked daily.

Focus groups were stratified to reflect research about racial differences in concerns about genetic research [29] and the differential prevalence of tobacco use by education [25, 28, 30].

### Procedure

Participants first provided informed consent and verified demographic and tobacco history information. Focus groups began with introductions and questions about reasons for smoking and the meaning of “genes” and “genetics.” These terms were then briefly explained in lay language to establish minimal common understanding [25, 28]. Participants shared their thoughts about possible links between genes, addiction, disease, and smoking, and then viewed a one-minute Associated Press news video (http://youtu.be/sO3X8xBr8YQ) describing the discovery of a genetic variant associated with severe nicotine addiction and increased likelihood of lung cancer [31].

Focus groups were facilitated by a race-matched moderator and note-taker. Moderators followed a semi-structured interview guide that incorporated broad open-ended questions crafted to be sufficiently flexible to elicit beliefs related to the a priori constructs of interest while allowing novel themes to emerge. Moderators probed for constructs of interest using a prepared script when these constructs failed to emerge organically. All questions and probes were phrased in non-academic language. The full interview guide has been published elsewhere [28].

### Data analysis

Audiorecordings were transcribed by a professional transcriber and checked for accuracy by a research assistant. The original data analysis process occurred from October 2012 to December 2013 using NVivo software [28, 32]. Discussions of additives were coded as “Additives” during the initial coding process but were not coded in-depth at the time because they were not of interest for primary analyses [25, 28]. Instead, these discussions were examined in the present data analysis for reasons described previously.

This targeted analysis (October 2015 to March 2016) used thematic analysis. SP listened to all audiorecordings and read all transcripts. SP and EW used an inductive and iterative process to develop, modify, and finalize an additive-specific codebook. After the codebook was finalized, SP coded the transcripts and EW verified that the quotes adequately represented the codes and that no possible codes were overlooked [33]. SP and EW met regularly throughout the coding process to identify and discuss major themes, to develop preliminary models describing the findings, and to refine the models based on re-examination of the codes, themes, and their interrelationships. EW provided feedback during all stages of the data analysis and interpretation process.

## Results

### Overview

Comments about cigarette additives arose without probing in 10 of 13 groups (3/4 HA, 3/4 LA, 1/2 HW, 3/3 LW). Specific examples included chemicals (e.g., “rat poison,” “ammonia,” “fiberglass,” and “formaldehyde, like the embalming fluid”) and biological contaminants (e.g., such as “rat turds” and “cow urine”).

In addition to specific examples, three broad themes emerged: (1) navigating the conceptual link between smoking and genetics, (2) providing an alternative mechanism of addiction, and (3) providing an alternative mechanism by which cigarette smoking exacerbates physical harm. The themes, described in detail below, did not seem to differ in content by education or race. Additive comments occurred at different times in each focus group.

Nearly all additives discussions were characterized by a pervasive and persistent tone of mistrust. Mistrust was expressed in several ways, including direct expressions such as “you can’t trust people these days,” expressions of uncertainty such as “you don’t know what they’re putting into the cigarettes,” and through the use of words heavily laden with negative affect such as “nasty” and “disgusting.” The speakers’ tones of voice, which manifested frustration, outrage, and indignation, also conveyed mistrust.

### Themes

#### Navigating conceptual link

In five groups (2 HA, 1 LA, 1 HW, 1 LW), additive discussions seemed to serve as a mechanism for navigating and understanding the conceptual link between smoking and genetics. Quotations related to this theme also demonstrate the tone of mistrust that was interwoven throughout the spontaneous discussions of additives.

One discussion began before the video was shown, in response to the moderator asking participants about their understanding of genetic risk and its relation to smoking:

[HA-Group2] Man 3: “Well, I feel like the tobacco companies, we don’t know what they put in cigarettes, you know, you really don’t. So they could put something in there to make a risk to the genetics.”

After watching the news story, that participant and another group member continued to process the novel genetic information by directly linking genetic risk to additives inserted into cigarettes by tobacco companies:

[HA-Group2] Man 3: “That’s what I was saying earlier about the tobacco companies… If there is a problem with the genetics and…the cigarettes created it…the tobacco companies caused [it].”

Woman 2: “Because you don’t know what they’ve – could have put in it.”

Man 3: “Right.”

Other participants contextualized the smoking-genetic link through additives in the environment and other consumer products. The following exchange occurred in response to a moderator’s question about whether participants believed the video’s report describing the genetic link to smoking.

[HA-Group1] Woman 1: “[E]verything is coming up with genetics, now. And it’s too much coming up… Even your food and things. So it just makes you wonder sometimes.”

Moderator: “So could that apply to smoking?”

Man 1 responds: “Yes! If it has to do with…the DNA or the biology of things, if you mix something in with something that’s there, then it alters it.”

These comments illustrate that participants did not consider a genetic risk related to cigarette smoking to be part of a smoker’s biology. They instead considered it to be a mutation spurred by the substances that tobacco companies, who participants mistrusted greatly, add to cigarettes. According to this view, in the absence of additives, there would be no genetic link to nicotine addiction.

#### Additives cause addiction

Participants in four groups (1 HA, 2 LA, 0 HW, 1 LW) blamed additives for addiction. These discussions were also steeped in mistrust:

[LW-Group2] Woman 1: “You never know what they’re puttin’ in [cigarettes] now…you don’t know what it is actually causing the addiction.”

[HA-Group1] Man 1: “Even with cigarettes…tobacco companies are putting everything into the tobacco…to make it more addictive.”

One group extended this idea, asserting that tobacco companies add substances for the express purpose of preventing people from quitting:

[LA-Group4] Woman 6: “I think they got something in there to make people keep – to keep their addiction…”

Woman 3: “There’s something in that cigarette, I can tell you.”

Woman 6: “It’s something in that cigarette.”

Not all participants who attributed addiction to additives mentioned nicotine by name. It is unclear whether they were unaware of the link between nicotine and addiction, or simply did not feel the need to name it, but the use of general terms such as “everything” and “something” in the quotations above suggests a lack of awareness. Nevertheless, a participant in one group used his beliefs about the addictive nature of additives to reject the news video’s assertion of a genetic link to nicotine addiction:

[LA-Group2] Man 2: “… [I]f you’re gonna serve tobacco then you serve tobacco. Do not extract [and reintroduce altered levels of] nicotine, do not put [in]…all them extra products…[T]he products they put in cigarettes, I believe, is the addiction. Not just the tobacco itself, which means that it’s not genetic…I think if they took all the products that they put in the cigarettes out, there would not be as many smokers as there is now.”

#### Additives exacerbate physical harm

Participants in seven groups (2 HA, 2 LA, 1 HW, 2 LW) partially attributed specific negative health consequences of smoking to additives, rather than to tobacco itself. The tone of mistrust was less pronounced here than in other themes:

[LW-Group1] Man 6: “Well, with all the chemicals that they put in cigarettes nowadays it is – smoking is *dangerous* [emphasis in original].”

[HW-Group2] Man 1: “I think there probably are additives to cigarettes that make it worse you know.”

Some participants followed the idea that additives (rather than tobacco) are harmful with a proposal that “natural” or additive-free cigarettes would be less harmful and therefore have less negative impact on health:

[HW-Group2] Woman 2: “I wonder how harmful additives are as compared to the tobacco itself…I wonder if…that might motivate people to – I don’t know – grow their own instead. Because if you are going to [smoke], I mean maybe there are ways that we could make it healthier or not as harmful.”

[LA-Group2] Woman 1: “I don’t think it’s the tobacco that’s killin’ me, it’s the – it’s the chemicals and stuff they put to it.”

These quotes demonstrate that most additive comments were grounded in the idea that additives and tobacco manufacturers – rather than tobacco itself – are the main culprits of intensifying the harms associated with cigarettes.

## Discussion

Participants spontaneously mentioned additives and other unknown substances in the context of discussing the smoking-genetic link. Additive comments appeared to serve various functions, including navigating the genetic link to smoking and blaming additives for the harms of tobacco use. Identifying specific well-known additives such as formaldehyde illustrated why additives were harmful and why tobacco companies should not be trusted.

Participants navigated the conceptual smoking-genetic link by citing additives as the perpetrator of genetic changes and contextualizing this link through additives in the environment and other consumer products. They imagined that smoking has a genetic component but felt that it is the substances added by tobacco companies that results in the genetic risk —not an inherent genetic element. Some participants argued that the presence of additives actually disproves the connection between genetics and smoking. For them, additives are the addictive substance and their existence in cigarettes demonstrates that addiction cannot be genetic.

These comments may suggest that some participants minimize the influence of genes on smoking behavior by asserting that external sources (e.g. tobacco companies) put additives in cigarettes and those additives lead to severe nicotine dependence, not the genes themselves. Participants used these statements to explain their behavior and, perhaps, deflect some responsibility for their addiction onto tobacco companies.

Using additives to justify dismissing the smoking-genetic link is consistent with other research demonstrating smokers’ skepticism about the validity of research establishing a smoking-genetic link [25, 28]. For some participants, accepting that their inherited DNA (an internal characteristic out of their control) may contribute to their behavior means diminished personal autonomy [25, 28, 34]. However, if genetic mutations are caused by additives (an external element out of their control) rather than inherited, they retain autonomy and validate their assertions that quitting only requires sufficient willpower (an internal characteristic within their control) [25]. This is consistent with research suggesting that people simultaneously seek to reduce feelings of personal blame and maintain a sense of control over their lives [35]. However, not all participants felt this way. Future research is needed to determine the extent to which smokers believe this connection.

The tendency for participants to blame additives for the harms of tobacco use and the assertion that “natural” cigarettes might be less harmful is consistent with research examining perceptions of “light” and “low tar” cigarettes [19] and a “no additives” campaign [36]. In our study, blaming additives for addiction might have helped participants to self-justify continued smoking despite acute awareness of the health consequences. However, there was insufficient data to determine if participants entirely attributed their continued smoking to additives, so additional research is needed.

Overall, participants were generally aware of the presence of additives in cigarettes and were able to name a few specific examples, but acknowledged many unknown substances are added to cigarettes. This awareness was steeped in mistrust of tobacco companies, as manifested through participants’ shared sense of uncertainty, regular use of accusatory language, indignant tones, and negative affective reactions towards additives. This is consistent with other research examining the public’s understanding and opinions of cigarette additives [11, 14, 16, 22], although those studies elicited participant reactions after providing information about additives.

Participants in our focus groups also used their knowledge and beliefs about additives to interpret new health information. This may lead to the use of ineffective harm reduction strategies (e.g., smoking “natural” cigarettes) and skepticism about novel public health campaigns that discuss the harms of tobacco use. Future studies should explore these reactions to better understand smokers’ opinions and behaviors. Such studies could inform communication strategies for intervention programs aimed at smoking cessation, which have shown great promise [37].

### Strengths, limitations, and future research

These results represent the first research gathered on smokers’ thoughts and beliefs about additives within the larger context of a study on genetics. Other studies examining beliefs about additives have purposefully elicited smokers’ reactions to information provided by the study team, whereas our results are drawn from spontaneous utterances in the context of a seemingly-unrelated study. This distinction can help further scientific understanding about how smokers think about the harms of smoking and their smoking behaviors. Additionally, smoking cessation campaigns could be designed to discuss additives and their actual role in smoking behavior and ability to quit. Furthermore, the *navigating the link* theme suggests that smoking counter-marketing efforts could highlight how additives might alter a person’s genetics or epigenome.

One strength of our study is that most participants (61%) were African American and people with less formal education (68%). This makes the results applicable to two demographic groups that disproportionately face difficulty quitting and have higher morbidity and mortality from smoking-related illnesses, especially cancer [38, 39].

This study should be interpreted in light of potential limitations. Results came from a secondary analysis of data for a study unrelated to smokers’ beliefs about additives. Although 10/13 groups mentioned additives, the same themes did not arise in all 10 groups. In groups where additive discussions occurred, no one provided contradictory views about these themes. However, lack of probes into these viewpoints limits our ability to draw definitive conclusions about participants’ beliefs about additives and their relationship to the themes we report. Lastly, while participants only referred to “additives” it is unclear if they knew the difference between constituents and additives and if this knowledge would have impacted beliefs.

Future studies could target these beliefs within other contexts, which could inform design of marketing and health communication campaigns. Although it is not possible to draw definitive conclusions about additives beliefs from this study alone, it does demonstrate that smokers’ thoughts about additives arise in a variety of conversational contexts, and it highlights avenues for future research. A future campaign might explicitly refute the idea that “no-additive” cigarettes are healthy, or that it is additives—not tobacco—that are addictive. Other campaigns might identify and highlight lesser-known additives or raise the salience of additives that elicit disgust. Additionally, these findings clearly highlight the need for public education around nicotine and addiction as well as about the constituents of cigarettes.

## Conclusions

Smokers in this study had distinct understandings about cigarette additives, which helped them deflect the health risks of smoking and understand the smoking-genetic link. Although mistrust may complicate communication about health risks of tobacco use, health communication experts could use smokers’ beliefs and feelings to design better anti-smoking messaging.

## Abbreviations

HA: Higher education/African American
HW: Higher education/White
LA: Lower education/African American
LW: Lower education/White
